# Anakinra treatment in patients with Familial Mediterranean Fever: a single-center experience

**DOI:** 10.1186/1546-0096-13-S1-P123

**Published:** 2015-09-28

**Authors:** S Ugurlu, B Ergezen, H Ozdogan

**Affiliations:** 1 Cerrahpasa Medical Faculty, University of Istanbul, Division of Rheumatology, Department of Internal Medicine, Istanbul, Turkey

## Background

Approximately 5 to10% of FMF patients do not respond and/or intolerant to to colchicine treatment. Several case reports and case series have pointed out the efficacy of IL-1 blockade in colchicine resistant FMF subgroup.

## Objectives

To review the patients followed in our center with FMF who received anakinra, an anti IL-1 receptor antagonist, because of insufficient colchicine response.

## Methods

FMF patients who were treated with anakinra were retrospectively reviewed with regard to indication, efficacy and adverse events. Patient global assessment was recorded before and throughout anakinra treatment.

## Results

There were 36 FMF patients with FMF who were treated with anakinra for various indications (amyloidosis in 11, colchicine resistant recurrent febrile attacks in 21, colchicine related side effects in 4). Two patients were excluded since they have been on anakinra for less than one month (one with amyloidosis, one pregnant). The mean age of the group was 34.8±10.8 years. The mean duration of the disease was 22.8 ±10,7 years. There were various co-existing pathologies among this study group like multiple sclerosis (1), ankylosing spondylitis (1), SLE (1), Behçet's disease (1), low grade lymphoma (1) and PAN (2). Five patients received anakinra during pregnancy. The mean colchicine dose was 2,09±0,49 mg/d. The mean duration of anakinra treatment was 13,34± 13,26 months. Twenty seven patients reported no attacks after the initiation of anakinra treatment whereas 5 patients reported at least 50% decrease in the attack frequency. Mean patient global assessment decreased from 8,74±2,2 to 1,74±2,6 under anakinra treatment (p=0.001).

Among the 9 patients with amyloidosis, anakinra was stopped in 2 patients because of increased proteinuria. However, a significant decrease in proteinuria was detected in 4 patients. Overall, 6 of our patients with amyloidosis are still on Anakinra treatment.

The treatment was stopped due to severe allergic reactions in 3 patients (severe disseminated rash in 1 and severe injection site reaction in 2) and because of infections in 2 patients (genital warts and urinary tract infection in 1 and sinusitis and folliculitis in 1). One of our patients reported that her psoriatic lesions got worse on anakinra. Twenty six patients reported no adverse events.

## Conclusions

Anakinra seems to be an effective and safe alternative in patients who have insufficient response to colchicine as well as in FMF related amyloidosis. The major cause of treatment termination is injection site reactions.

